# Extensive Portal Vein Thrombosis Following Laparoscopic Cholecystectomy: Interplay of Liver Cirrhosis, Psoriasis, and Anticoagulation Oversight

**DOI:** 10.7759/cureus.79958

**Published:** 2025-03-03

**Authors:** Merry A Mathew, Kent-Andrew Boucher

**Affiliations:** 1 School of Medicine, Texas Tech University Health Sciences Center, Lubbock, USA; 2 Department of Surgery, Texas Tech University Health Sciences Center, Odessa, USA

**Keywords:** acute portal vein thrombosis, alcoholic liver cirrhosis, hypercoagulable state, plaque psoriasis, robot-assisted laparoscopic cholecystectomy

## Abstract

Portal vein thrombosis (PVT) is a rare but serious condition often associated with liver cirrhosis, malignancy, and hypercoagulable states. While PVT following laparoscopic surgery is uncommon, certain risk factors can predispose patients to this complication. A 65-year-old male with a history of untreated plaque psoriasis and alcoholic liver cirrhosis with portal hypertension developed PVT after a robot-assisted laparoscopic cholecystectomy. The patient presented with abdominal swelling and mild epigastric pain two months postoperatively. Imaging revealed an occlusive thrombus in the portal vein extending into the splenic and superior mesenteric veins, accompanied by bowel congestion, edema, and early signs of ischemia. The patient had been on warfarin prior to surgery for an unclear reason but had failed to resume warfarin use postoperatively due to a lack of follow-up with his primary care physician. After managing the patient's PVT with a heparin drip followed by apixaban, his symptoms improved. A thrombophilia panel revealed decreased activity of antithrombin III, factor II, and protein C. Thorough preoperative history-taking and postoperative primary care follow-up are essential, particularly in patients with multiple thromboembolic risk factors. The unexpected role of untreated psoriasis as a potential contributor to PVT highlights the need for further research into the systemic effects of chronic inflammatory conditions.

## Introduction

Portal vein thrombosis (PVT) is a rare condition with varying incidence and prevalence rates reported in the literature. A large population-based study in southern Sweden found the prevalence of PVT to be 1%. Of those patients with PVT, 28% had liver cirrhosis, 23% had primary hepatobiliary malignancy, 44% had secondary hepatobiliary malignancy, 10% had major abdominal infectious or inflammatory disease, and 3% had a myeloproliferative disorder [1]. The prognosis of PVT mainly depends on the patient’s co-morbid conditions, especially cirrhosis or malignancy. The one-year and five-year overall survival rates are 69% and 54%, respectively. However, in the absence of malignancy and cirrhosis, the survival rates improve to 92% at one year and 76% at five years [2].

Local risk factors for PVT include hepatopancreaticobiliary malignancies, cirrhosis, intra-abdominal surgery, trauma, and inflammatory abdominal conditions. Systemic risk factors include inherited thrombophilias, myeloproliferative disorders, pregnancy, and oral contraceptive use [3]. Notably, PVT appears to be strongly associated with severe cirrhosis and hepatocellular carcinoma [4,5]. Asymptomatic acute PVT is often found incidentally. Patients with symptomatic PVT commonly present with nonspecific symptoms, including abdominal pain, diarrhea, nausea, vomiting, abdominal distention, gastrointestinal bleeding, fever, sepsis, and lactic acidosis [3].

When PVT is suspected, color Doppler ultrasonography (CDUS) is the first-line approach for diagnosis, with a sensitivity of 89% and a specificity of 92% [6]. If the portal venous system cannot be visualized by ultrasound or if the extent of thrombotic involvement in the splenic and mesenteric veins cannot be adequately assessed, magnetic resonance angiography (MRA) is the next best step in the diagnostic approach. MRA has a sensitivity of 100% and a specificity of 98% in the diagnosis of PVT [7]. Computed tomography (CT) with enhanced contrast can also show a PVT and is useful in differentiating between benign and malignant PVT in patients with cirrhosis [8].

Anticoagulation therapy is generally recommended for patients with cirrhotic PVT to prevent thrombus progression and potential complications. Anticoagulant options typically include unfractionated heparin, low-molecular-weight heparin (LMWH), or warfarin, though direct oral anticoagulants (DOACs) are showing promise in small studies [9]. For patients with non-cirrhotic PVT, systemic anticoagulation with LMWH is the primary treatment, especially if the clot is recent [9]. A minimum of six months of anticoagulation is recommended if the predisposing factor has been reversed. For patients with thrombophilias, lifelong anticoagulation may be necessary. In cases of acute PVT with complications like imminent bowel infarction, endovascular thrombolysis or surgical intervention may be required [9]. In this report, we present a case of PVT after robot-assisted laparoscopic cholecystectomy in a 65-year-old male with multiple risk factors for venous thromboembolism.

This article was previously presented as a poster presentation at the North Texas Chapter of the American College of Surgeons meeting on February 22, 2025.

## Case presentation

A 65-year-old Hispanic male presented to the emergency department with a two-day history of right upper quadrant and epigastric abdominal pain. The patient reported no past medical history, no surgical history, no use of home medications, and denied alcohol, tobacco, and illicit substance use at this time. Since this patient reported no comorbidities, routine preoperative workup was conducted, which involved a complete blood count (CBC), comprehensive metabolic panel (CMP), imaging, and a urinalysis. CBC and CMP revealed leukocytosis and a slightly elevated anion gap. To rule out acute pancreatitis, a lipase level was obtained, which was within normal limits (Table 1). CT scan of the abdomen revealed a distended gallbladder with gallstones and pericholecystic stranding, consistent with acute cholecystitis. Urinalysis was within normal limits. On physical examination, the patient exhibited significant tenderness to palpation in the right upper quadrant with a positive Murphy’s sign. He was diagnosed with acute cholecystitis and admitted for robot-assisted laparoscopic cholecystectomy.

**Table 1:** Pertinent findings from preoperative laboratory results.

Lab test	Value	Reference range	Notes
White blood cell count	19.2 × 10⁹/L	4.0-11.0 × 10⁹/L	Elevated (leukocytosis)
Anion gap	13	8-12 mmol/L	Mildly elevated
Lipase	14	10-140 U/L	Within normal limits

The patient was taken to the operating room on hospital day one, where his gallbladder was noted to be necrotic and extensively friable, with bleeding from all surfaces. After careful dissection and irrigation, the gallbladder was removed, and hemostasis was achieved with electrocautery. His liver was noted to be markedly macronodular, consistent with cirrhosis. Two liver core biopsies were collected for pathological evaluation, and a 19 French Jackson-Pratt (JP) drain was placed in the right subhepatic recess. Pathological evaluation of the liver core biopsies revealed liver cirrhosis with stage four fibrosis.

On postoperative day two, the patient had a violent cough, after which his JP drain developed sanguineous output. He had bleeding around his JP drain that was unresponsive to 15 minutes of direct pressure. The patient remained hemodynamically stable during the entire episode. Laboratory tests for hemoglobin, prothrombin time (PT), international normalized ratio (INR), and activated partial thromboplastin time (aPTT) were ordered to evaluate the status of acute blood loss anemia and coagulation function. Laboratory values (Table 2) suggested severely impaired coagulation function, which likely contributed to bleeding at the surgical site. Two units of fresh-frozen plasma, one unit of packed red blood cells, and vitamin K were administered. Bleeding resolution and hemoglobin stabilization were achieved over the next few days. Upon further discussion with the patient, it was discovered that he had been taking 4.5 mg of warfarin daily for many years. He had failed to disclose his warfarin use preoperatively and was unsure of the reason for his warfarin use. The patient was discharged on postoperative day four with instructions to follow up with his primary care provider regarding warfarin use and to discontinue warfarin until then. He did not follow up with his primary care provider as directed.

**Table 2:** Laboratory values on postoperative day two. aPTT: activated partial thromboplastin time; PT: prothrombin time; INR: international normalized ratio.

Lab test	Value	Reference range	Notes
Hemoglobin	12.6 → 7.7 g/dL	14-18 g/dL (male)	Significant drop over 24 hours
aPTT	71 sec	25-35 sec	Prolonged
PT	55 sec	11-13 sec	Prolonged
INR	6.3	0.8-1.2	Elevated

**Figure 1:**
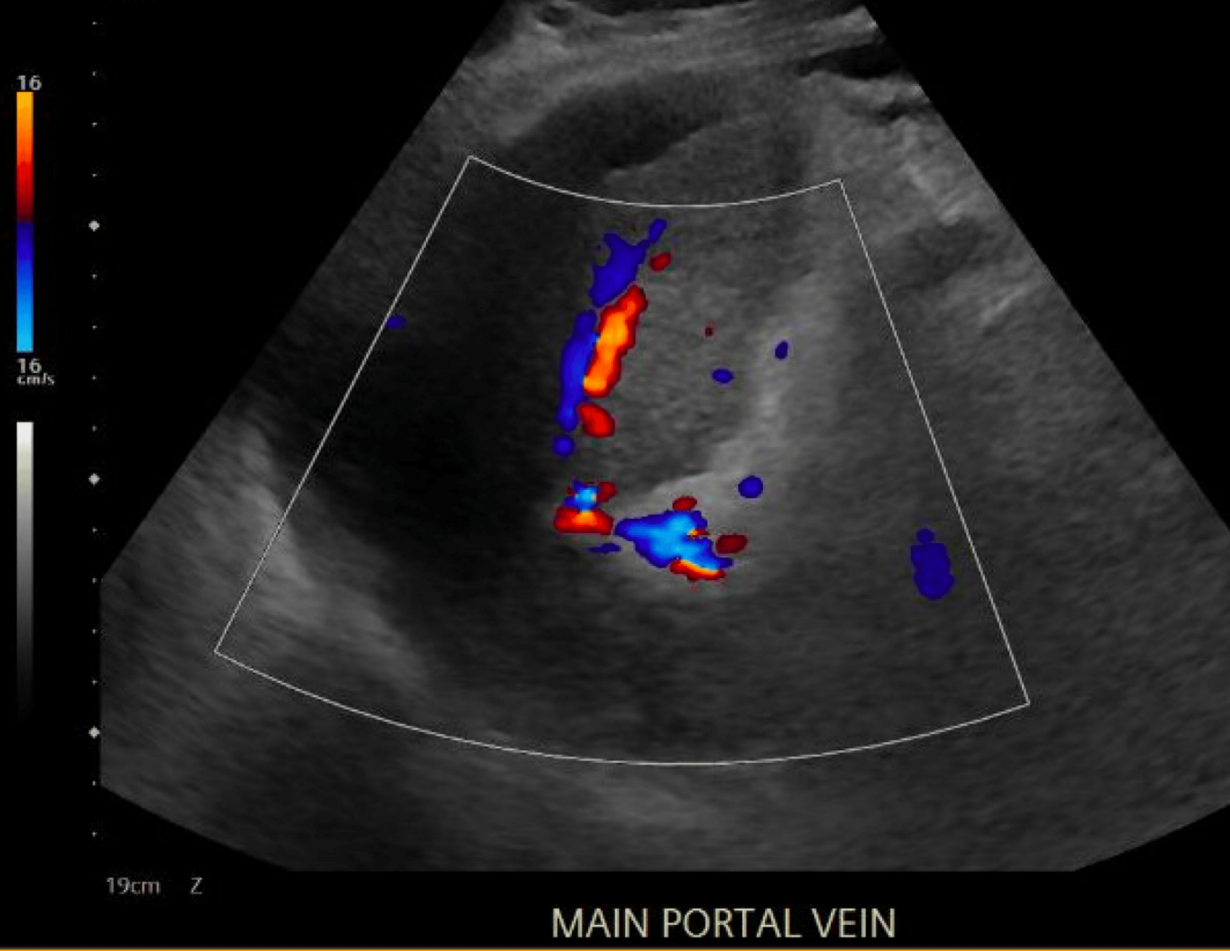
CDUS showing limited abnormal visceral Doppler of the main portal vein, indicating biphasic flow and/or thrombus with cavernous transformation. CDUS: color Doppler ultrasonography.

Two months later, the patient returned to the emergency department with a one-week history of abdominal swelling and progressively worsening mild epigastric pain. He denied associated fever, chest pain, shortness of breath, nausea, vomiting, diarrhea, or constipation. His past medical history was significant for alcohol use disorder in remission for six months and liver cirrhosis with portal hypertension. At this visit, the patient was noted to have extensive plaque psoriasis covering his abdomen, back, and upper and lower extremities, which he reported as untreated. His venous lactate level was elevated at 2.6 mmol/L. CDUS showed limited abnormal visceral Doppler of the portal vein, indicating biphasic flow and/or thrombus with cavernous transformation (Figure 1).

**Figure 2:**
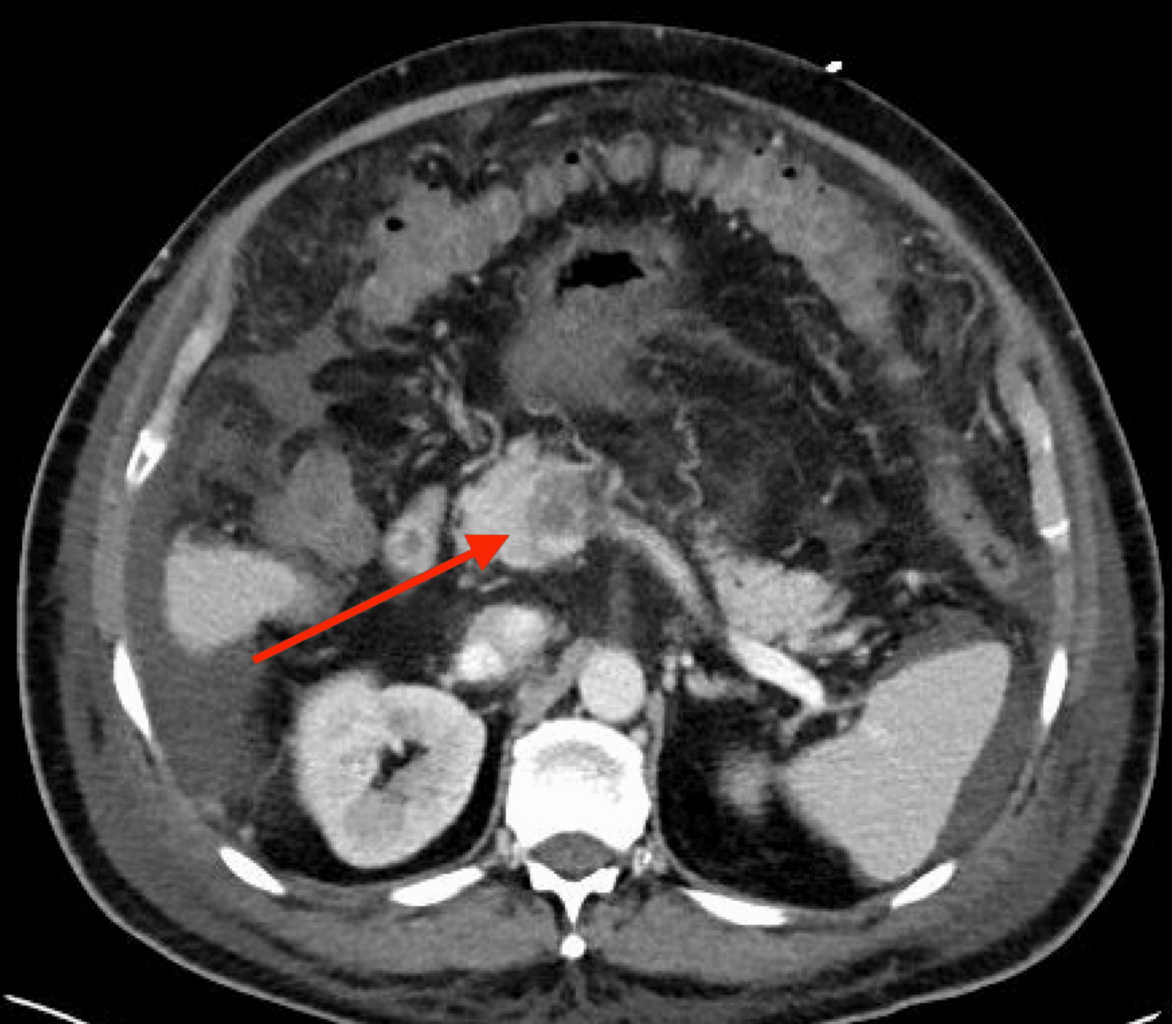
CT of the abdomen (axial) demonstrating portal vein thrombus.

**Figure 3:**
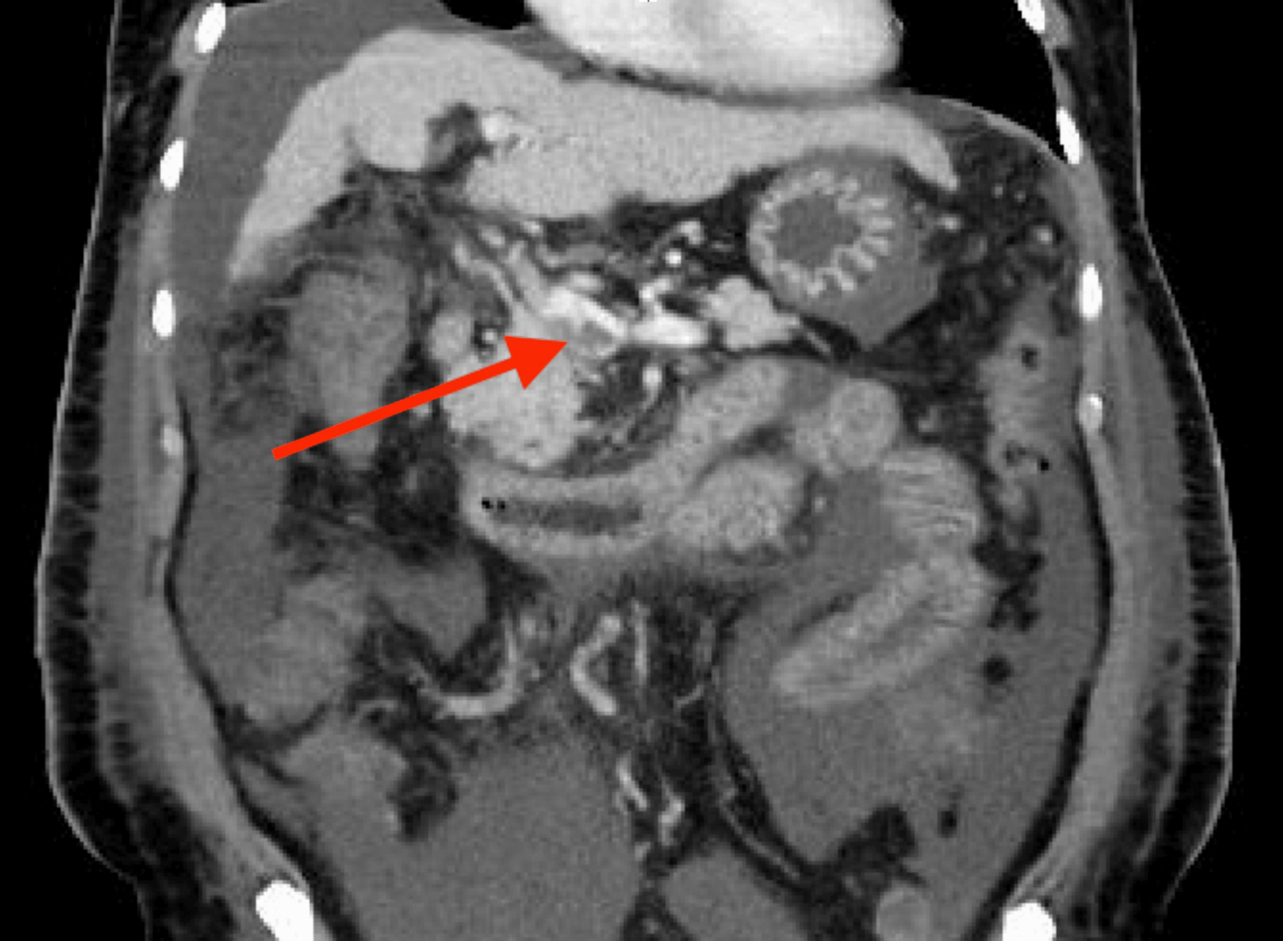
CT of the abdomen/pelvis (coronal) demonstrating portal vein thrombus.

CT of the abdomen/pelvis showed occlusive portal vein thrombus extending into the proximal splenic vein and throughout nearly the entirety of the superior mesenteric vein (Figures 2, 3). Additional findings included severe edema, congestion, and radiographic signs of early ischemia of the mid to distal jejunum due to venous congestion from superior mesenteric vein thrombosis.

**Figure 4:**
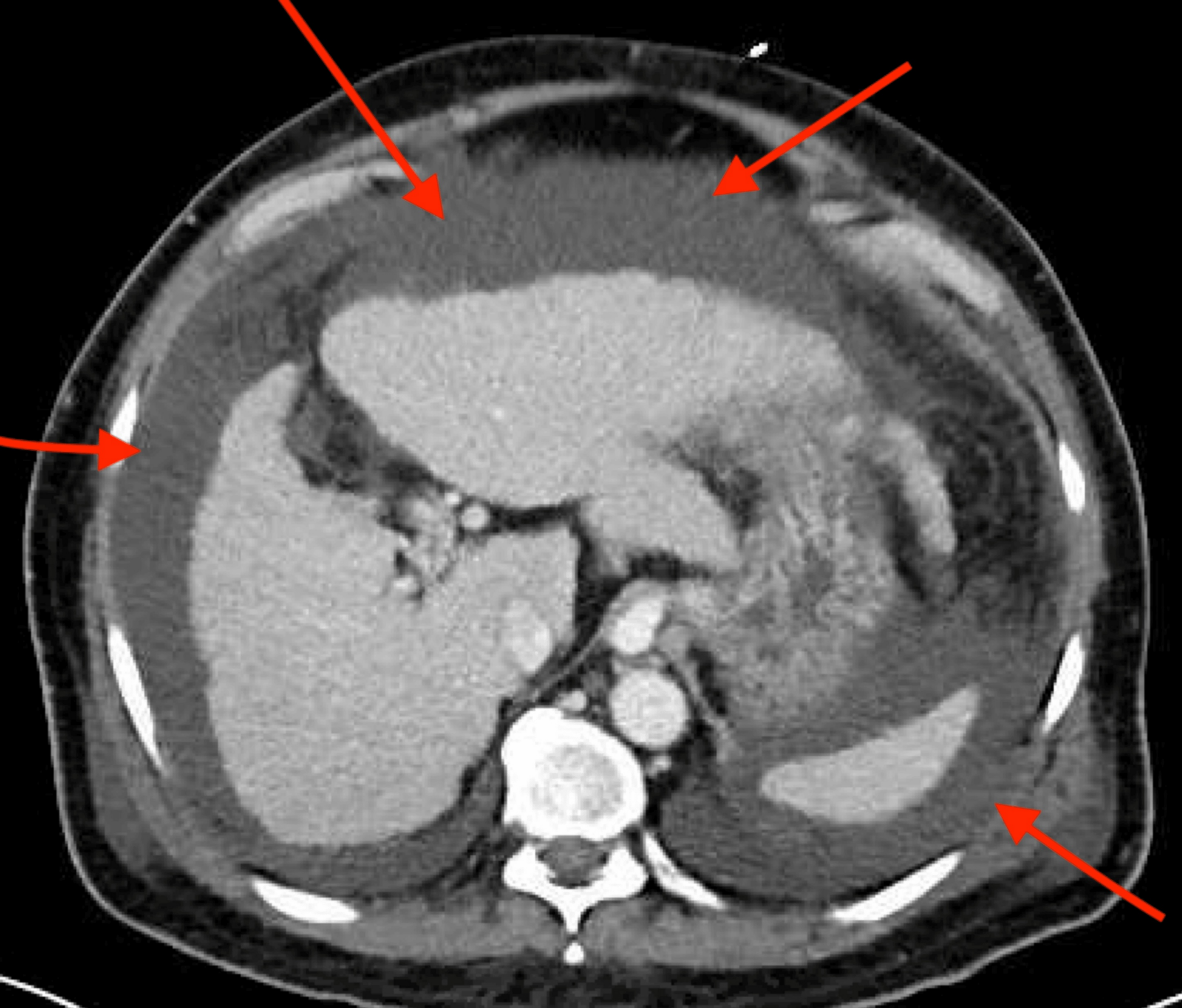
CT of the abdomen (axial) showing severe abdominal ascites.

There was thickening and congestion in the ascending colon, which was suspicious for congestive/ischemic changes. A small cirrhotic liver and moderate to large volume abdominopelvic ascites were also visualized, indicating decompensated portal hypertension (Figure 4).

It was discovered that the patient had not resumed his warfarin therapy following his cholecystectomy. Given the patient's significant co-morbidities, including large esophageal varices and decompensated portal hypertension, he was deemed at high surgical risk for catheter-directed thrombolysis. Due to the limited benefits of transjugular intrahepatic portosystemic shunt for PVT, the patient was managed medically with a heparin drip for two days and then transitioned to apixaban, a DOAC. The patient was discharged on hospital day six with improved abdominal pain and distension and was prescribed apixaban for long-term outpatient anticoagulation. A thrombophilia panel was performed, including tests for factor V Leiden mutation, protein C activity, antithrombin III activity, and factor II activity. Results revealed decreased activity of antithrombin III, factor II, and protein C (Table 3). Factor V Leiden genetic testing was negative. The patient was directed to follow up with a hematologist for further workup.

**Table 3:** Thrombophilia panel demonstrating decreased antithrombin II, factor II, and protein C activity. N/A: not applicable.

Lab test	Value	Reference range	Notes
Antithrombin III activity	49%	80-120%	Decreased
Factor II activity	68%	70-120%	Slightly decreased
Factor V Leiden mutation	Negative	N/A	No mutation detected
Protein C activity	63%	70-150%	Slightly decreased

## Discussion

This patient presented with multiple risk factors for PVT, including liver cirrhosis, recent abdominal surgery, and psoriasis. The presence of cirrhosis leads to stasis of flow in the portal vein, creating a prothrombotic environment. Additionally, cirrhosis leads to an imbalance between procoagulant and anticoagulant factors, further contributing to hypercoagulability [10].

PVT is a recognized but rare complication of laparoscopic procedures, likely resulting from a combination of local factors, such as inflammation and trauma to the portal venous system, and systemic factors, including thrombophilias and other causes of hypercoagulability. PVT is an uncommon occurrence after laparoscopic cholecystectomy, with only six cases reported in the English-language literature. In these cases, the identified factors associated with a hypercoagulable state included low protein S levels, prothrombin G20210A mutation, dengue viral infection, elevated anticardiolipin antibodies, and oral contraceptive use [11-15]. One case had no identifiable risk factors [16].

The patient's history of extensive, untreated psoriasis could have played a role in the development of PVT. Psoriasis is increasingly recognized as a systemic inflammatory condition that extends beyond skin involvement. One nationwide Danish cohort study found that psoriatic patients are at an increased risk for venous thromboembolism [17]. Chronic inflammation associated with psoriasis can lead to endothelial dysfunction and a hypercoagulable state. This systemic inflammation activates platelets, increases levels of procoagulant factors, and impairs fibrinolysis, all of which contribute to an increased risk of thrombotic events [18].

Additionally, the patient was on warfarin for an unknown coagulation issue. His hematologic workup showed decreased activity of antithrombin III, factor II, and protein C. In a patient with liver cirrhosis, these findings are expected since the liver synthesizes these proteins [10]. However, an underlying genetic cause for these derangements cannot be ruled out. He did not have a factor V Leiden mutation. Given the inconclusive nature of the initial workup, further testing is warranted to investigate other potential causes of hypercoagulability, such as prothrombin G20210A mutation, methylenetetrahydrofolate reductase gene mutations, protein S deficiency, JAK2 V617F mutation, and antiphospholipid antibody testing. Although there is no consensus on genetic screening recommendations for cirrhotic PVT, one study found a thrombophilic genotype in 69.5% of cirrhotic patients with PVT, suggesting a potential role for screening for inherited thrombophilia in these patients [19].

This case underscores the critical importance of thorough preoperative evaluation, medication stewardship, and postoperative primary care follow-up, particularly for patients with multiple risk factors for venous thromboembolism. The underlying cause for the patient's warfarin use was initially missed, and the reason for its use remains unclear, underscoring the importance of obtaining a comprehensive medical history during the preoperative period to prevent complications. In this case, the patient withheld certain information during the preoperative evaluation, potentially due to cultural or social factors. To address this in the future, healthcare providers build trust and rapport to encourage open communication by using culturally sensitive communication, employing active listening skills, and demonstrating empathy for the patient's perspective and experiences. The decision to discontinue warfarin postoperatively was appropriate. However, the patient experienced a prolonged period without anticoagulation. Proper transition of care and postoperative follow-up can significantly reduce the risk of complications and readmissions. One retrospective cohort study found that patients who had a follow-up visit with a primary care physician within 30 days of discharge for an emergency general surgery condition had a lower risk of readmission compared to those who did not [20]. The challenging nature of this case, marked by multiple risk factors for PVT, patient noncompliance with follow-up, and an unclear medical history, further emphasizes the complexity of managing such patients.

## Conclusions

This case highlights a 65-year-old male with multiple risk factors for PVT, including decompensated liver cirrhosis, recent laparoscopic cholecystectomy, and untreated plaque psoriasis. The patient presented with abdominal swelling and pain, which led to the discovery of an occlusive portal vein thrombus complicated by bowel edema, congestion, and ischemia. The development of PVT following laparoscopic cholecystectomy, although rare, emphasizes the need for heightened awareness and early diagnosis, especially in patients with predisposing factors. Psoriasis, a systemic inflammatory condition, may play a role in the development of venous thromboembolism. The prolonged period without anticoagulation in this case underscores the importance of postoperative primary care follow-up, particularly in patients with a history of anticoagulant use. Genetic screening for underlying causes of hypercoagulability may be warranted in similar cases.
